# Glassware design and drinking behaviours: a review of impact and mechanisms using a new typology of drinking behaviours

**DOI:** 10.1080/17437199.2020.1842230

**Published:** 2020-11-18

**Authors:** Tess Langfield, Rachel Pechey, Mark A. Pilling, Theresa M. Marteau

**Affiliations:** Behaviour and Health Research Unit, Department of Public Health and Primary Care, University of Cambridge, Cambridge, UK

**Keywords:** Choice architecture, glassware design, drinking, micro-drinking behaviours, affordance, perceptual effects

## Abstract

Much of the global burden of disease is attributable to unhealthy behaviour, including excessive consumption of alcohol and sugar-sweetened beverages. Developing effective methods to change these drinking behaviours could inform policies to improve population health. In line with an increasing interest in environmental-level interventions – i.e., changing the environment in which a behaviour occurs in order to change the behaviour of interest – this review first describes the existing evidence of the impact of glassware design (including capacity and shape) on drinking behaviours (e.g., at the ‘micro’ level – including sip size, as well as at the macro level – including amount consumed). The roles of two sets of possible underlying mechanisms – perception and affordance – are also explored. Finally, this review sets out a provisional typology of drinking behaviours to enable more systematic approaches to the study of these behaviours. While there is a paucity of evidence – in particular on measures of consumption – this growing evidence base suggests promising targets for novel interventions involving glassware design to reduce the consumption of drinks that harm health.

**Trial registration:** ISRCTN10456720.

## Changing drinking behaviour to prevent disease

Much of the global burden of disease is attributable to several unhealthy behaviours, including excessive consumption of alcohol and sugar-sweetened beverages (Chazelas et al., 2019; Singh et al., 2015; Stanaway et al., 2018; WHO Global Status Report on Alcohol and Health, 2018). Alcohol alone is linked to over sixty different health conditions (Room et al., 2005), and the consumption of sugar-sweetened beverages is associated with obesity, type 2 diabetes, cardiovascular disease, and a number of other health conditions (e.g., Malik et al., 2010; Scientific Advisory Committee on Nutrition, 2015; Te Morenga et al., 2012). Measures aimed at reducing the intake of alcohol and sugary drinks are thus high on national and international government agendas (e.g., Department of Health, 2016a, 2016b; U.S. Department of Health and Human Services and U.S. Department of Agriculture, 2015; World Health Organisation, 2015).

## Changing behaviour by changing cues in physical environments

It has been suggested that effective interventions for changing routine or habitual behaviours should acknowledge the important role of automatic, non-conscious processes in shaping these behaviours (e.g., Hagger, 2016; Hollands et al., 2016; Marteau, 2018; Marteau et al., 2012). One approach to changing behaviour which is thought to target these automatic processes is *choice architecture,* also known as *nudging.* Here, choices, environments, or cues within environments are designed to elicit a change in behaviour, often outside of awareness. The concept of ‘nudging’ was popularised by Richard Thaler and co-author Cass Sunstein in their 2008 book ‘Nudge: Improving decisions about health, wealth and happiness’ (Thaler & Sunstein, 2008). Though this approach has gained traction among researchers and policymakers in recent years, similar ideas about human behaviour can be traced to the end of the nineteenth century, when William James (1899) wrote that ‘ninety-nine hundredths, or possibly, nine hundred and ninety-nine thousandths of our activity is purely automatic and habitual’ (p.65). Later, the Behaviourism paradigm that dominated mid-twentieth century Psychology held the environment or situation as central in determining behaviour (e.g., Skinner, 1974), and couched behaviour in its environmental context (Blackman, 1985). Indeed, in an early semi-naturalistic study investigating factors influencing drinking, Rosenbluth et al. (1978) found that the setting in which drinking takes place (including the characteristics and numbers of companion drinkers) influenced drinking behaviours including amount consumed and drinking rate. The authors suggested that contextual factors were at least as important in driving drinking as the characteristics of the drinker and their drinking history, in line with the ‘widespread behavioural view’ (p.120).

With the recent popularisation of interventions to change behaviours via changing cues in environments or *nudging*, a lack of clarity in the definitions of key concepts and terms has become apparent (e.g., Marchiori et al., 2017). In response to this lack of clarity, a Typology of Interventions in Physical and Proximal Micro-Environments (TIPPME) has been developed (Hollands et al., 2013) and refined (Hollands et al., 2017) for use as a framework for conceptualising physical environment interventions. Such interventions include altering the placement or properties of products, associated objects, and the wider environment in which the products exist, in order to change behaviours.

## Glassware as a cue to consume

One property of the physical, proximal micro-environment that may influence consumption is the drinks container. Although many foods can be consumed directly – such as fruit, biscuits, and sandwiches – drinks are almost always consumed from some form of drinking vessel. Thus, the drinks container can be seen as a mediator of drinking (Spence & Wan, 2015). Drinks containers take many forms, varying with type of drink – e.g., beer *vs* coffee – and drinking context – e.g., picnic *vs* formal banquet. The focus of this review is on both alcoholic and non-alcoholic drinks, and glassware in the form of glasses and cups *–* but not cans or bottles – consumed in any drinking context.

Glassware can take many different forms and designs including different capacities and shapes. It has been suggested that design of a glass – including its size and shape – has become ‘an integral part of marketing activity’, warranting careful consideration to maximise sales (Stead et al., 2014, p. 318). Indeed, wine glasses have increased in capacity over the last three hundred years and particularly since the 1990s when their size has almost doubled, likely contributing to the increase in wine consumed over the last thirty years (Zupan, Evans, et al., 2017). Given that the number of people drinking wine was roughly constant over this period, increased wine glass size is a good candidate for understanding the increase in consumption (‘British wine glasses have got bigger over the years’, 2017, para 4), though changes in number of drinks consumed and the amount consumed per drinking occasion could also play a role. Due to the potential impact of glassware design on drinking behaviour and outcomes, glassware design is a target for reducing consumption of health-harming drinks. The primary aim of this review is therefore to examine the existing evidence on the impact of glassware design on drinking behaviours and outcomes.

## Potential mechanisms: perception and affordance

**Figure 1.**
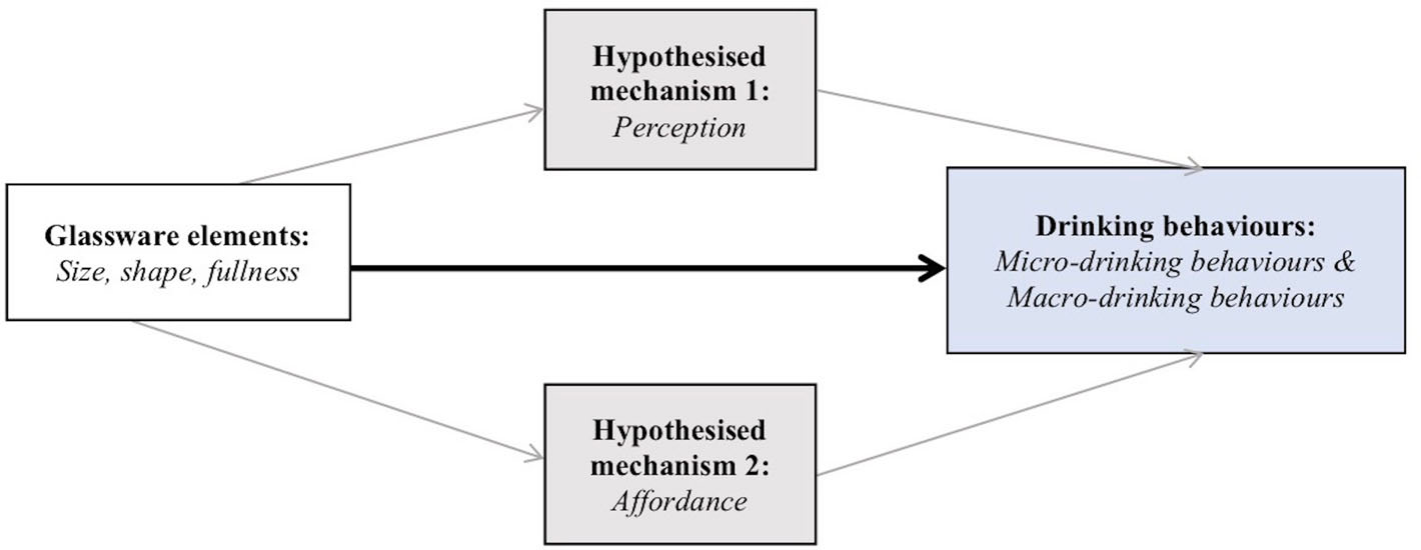
Logic model to organise evidence on the impacts of drinks containers on micro- and macro- drinking behaviours, with two proposed mechanisms: perception and affordance.

To further understand and optimise any potential effects of glassware design on drinking, and to facilitate the design of effective interventions, it is helpful to conceptualise potential ‘mechanisms of action’ (Michie et al., 2018). This is related to the ‘experimental medicine’ approach, which highlights the importance of understanding not only *whether* a behaviour change intervention is effective, but how it works to change behaviour (e.g., Sheeran et al., 2017). Figure 1 illustrates two – neither exhaustive nor exclusive – potential mechanisms which have been highlighted by research to date as factors that may mediate or contribute to the effects of glassware design on drinking behaviours. First, there may be *perceptual effects* of glassware design. In this review, as in previous papers (e.g., Spence & Wan, 2015), perceptual effects of glassware design will include subjective judgments (e.g., liking for drinks and other subjective responses), as well as visual judgments (e.g., visual perceptions of liquid volume). Second, there may be *affordance* by glassware design. This relates to the observation that some glasses, by dint of some feature of their design, appear to invite or afford faster drinking rates, larger gulps, or other patterns of behaviours that may, in turn, influence how much is drunk from them. The penultimate aim of this paper is to outline and evaluate evidence in support of these proposed mechanisms.

## Defining drinking behaviours

‘Drinking behaviour’ is a broad term, encompassing a number of behaviours. The primary aim of this paper is to review the effects of glassware design on measures of consumption – the key outcome of interest to researchers and policymakers interested in reducing consumption of health-harming drinks and increasing consumption of healthy drinks. When organising and discussing the evidence in relation to the primary aim, it is important to distinguish between larger-scale (*macro*) drinking behaviours – amount consumed and proxies for it – and smaller-scale (*micro*) drinking behaviours – the micro-structure of drinking behaviours such as sip size, and the evidence is organised as such. On one level, micro-drinking behaviours are a fundamental feature of drinking: any drinking episode can be characterised by different micro-drinking elements, for example, how large the sips were, how many sips were taken, and whether pace of consumption was consistent over time. On another level, these micro-drinking behaviours might be seen as proxies for, or predictors of, larger-scale ‘macro’ drinking behaviours such as volume consumed. Thus delineating drinking behaviours by contrasting micro- with macro- behaviours can uncover the mechanics of *how* effects on consumption might work, which may yield new insights beyond what is captured from studying consumption outcomes alone. In the absence of an existing typology, the final aim of this paper will be to present a provisional typology of micro- and macro- drinking behaviours on the basis of the existing evidence, to enable more systematic study and better reporting of these behaviours in future studies.

## Aims

1. To summarise evidence of the impact of glassware design – notably size, shape, and resulting fullness – on macro-drinking behaviours.
2. To summarise evidence of the impact of glassware design – notably size, shape, and resulting fullness – on micro-drinking behaviours.
3. To describe two potential mechanisms through which glassware design might impact on drinking behaviours, namely perception and affordance.
4. To develop a preliminary typology of drinking behaviours

## Search strategy

An electronic literature search was completed on 2 Sep 2020, to source relevant papers on the impact of glassware design (size, shape, fullness) on macro- and micro- drinking behaviours (i.e., aims I and II). MEDLINE and PsycInfo databases were used. Eligibility criteria included: experimental design (non-observational or literature reviews), measuring human drinking behaviour (not measured virtually, online, or using self-reported drinking), with researcher assignment to condition (e.g., glassware design features manipulated, not participant self-selected, for between-subject designs) or presentation order (for within-subject designs).

The following search terms were used for each database: (drink* OR drunk* OR consum* OR sale* OR sold OR purchas* OR sip* OR taste* OR pour* OR drink frequency OR drink number OR number of drinks) AND (glass* OR drinkware OR cup OR container) AND (size* OR capacit* OR portion* OR volume* OR shape* OR fullness).

Electronic database searching returned 671 papers (607 after removing duplicates). Twenty-three papers met the eligibility criteria. Snowball searching and personal communications revealed an additional 4 papers, providing a total of 27 papers included for review. For details of all included studies, see Supplementary Information.

## I. Impacts of glassware design on macro-drinking behaviours

Searches were conducted for experimental studies manipulating glassware (size, shape, and fullness) and measuring human drinking behaviour (amount consumed, amount purchased, amount poured, and number of drinks), for alcoholic and non-alcoholic drinks. Observational studies and literature reviews were excluded. Studies were only included if they measured drinking behaviours (not online or via self-report).

### i. Amount consumed

Four studies have examined the impact of glassware design on amount consumed. Kersbergen et al. (2018) investigated whether reducing the serving size of alcohol could reduce alcohol consumed (measured in UK units) in a semi-naturalistic laboratory setting. Pairs of participants were offered beer, cider, or wine in either standard or reduced serving sizes. Findings suggested that by reducing the serving size of alcohol by 25%, alcohol units consumed were reduced by ∼20%. However, though the intended manipulation was portion size, in order to keep glass fullness constant, glass sizes also varied with portion size. As a result, the reduced consumption which was observed may have been caused by reduced portion size, reduced glass size, or a combination of the two variables.

In a follow up study, Kersbergen et al. (2018) investigated whether reducing the serving size of alcohol reduced the volume of alcohol consumed – expressed in units (UK definition) of alcohol – in a bar setting. Again, participants were offered beer, cider, or wine in either standard or reduced serving sizes, with the price of drinks proportional to the serving size. The primary outcome measure was amount of alcohol consumed (expressed in UK units) within 3 h of observation. Consumption was measured through covert observation of drinking in the bar by researchers – in particular by counting the number of beverages consumed at each table (given known serving sizes), and through counting the number of beverages sold (minus wastage). Findings suggested that by reducing the serving size of alcohol, by ∼30%, alcohol units consumed were reduced by ∼35%. As before, given that glass size varied with serving size, it is possible that the reduction in intake found was in part due to the reduction in glass size.

Two studies have reported on the impact of glass shape on amount consumed. Raghubir and Krishna (1999) compared amount of a soft drink consumed when served in a taller and wider glass of identical capacities, finding more was consumed from the taller glasses. More recently, Langfield et al. (2020) compared consumption of soft drinks served in straight-sided wine flutes and outward-sloped martini coupes, during a 10 min bogus taste test. They found that when tasting and rating drinks served in straight-sided flutes, participants consumed 72 ml less overall than when sipping from outward-sloped flutes.

### ii. Amount purchased

Nine studies report on the impact of glassware design on amount of drinks purchased. In the first of a series of studies, Pechey et al. (2016) investigated the impact of wine glass size on sales of wine in a bar/restaurant setting. In this study, as in all the following studies in this section, drinking was not directly measured, with purchasing of wine for immediate consumption used as a proxy for actual consumption. Wine sales increased by 9.4% when sold using larger glasses (370 ml), as compared with standard glasses (300 ml), with no differences in sales observed when using smaller (250 ml) glasses compared with standard glasses (300 ml). Six follow up studies have been conducted in bars and restaurants (Clarke et al., 2019; Pechey et al., 2017), summarised in a mega-analysis by Pilling et al. (2020), the results of which will be reported here (but see Supplementary Information for further details on each study). This analysis indicated that, when combining all data, there were no effects of wine glass size on sales of wine in bars. However, in restaurants, compared with the 300 ml glass, wine sales were 7% higher when 370 ml glasses were used. There was also a trend to suggest that wine sales decreased by around 10% from smaller (250 ml) glasses, though this was not significant.

Using a similar design, Troy et al. (2015) compared pub sales between weekends when straight-sided *vs* outward-sloped beer glasses were used. Though the primary aim of this field study was to assess feasibility, the authors noted that sales were 24% lower when beer was served using straight compared with curved glasses. This finding awaits replication in a larger field study, currently underway in 24 bars (see Brocklebank, 2019 for trial pre-registration).

### iii. Amount poured

Another possible proxy for consumption, particularly for drinking at home where drinks are typically self-served rather than served by staff as in a commercial establishment, is amount poured. Nineteen studies have investigated the effect of glassware design (size and shape) on amount poured, including 4 which measure the free pouring of self-defined drinks (e.g., ‘typical serving’), 14 which stipulate an exact amount to be poured (e.g., a ‘standard drink’ serving of alcohol), and 1 which measures both.

In two field studies measuring volume poured in freely poured self-servings for subsequent consumption, Wansink and Van Ittersum (2003) found between 19% and 74% more juice was poured into short-wide glasses than tall-narrow ones (capacity both 659 ml). In a laboratory study measuring self-defined pours of alcohol (not for consumption), Knibb et al. (2018) found no evidence that short-wide *vs* tall-narrow glasses differed in terms of amount poured. It is worth noting here that variation in ‘self-defined’ servings might contribute to the absence of an effect of glass shape on poured volumes in between-subjects designs (such as Knibb et al., 2018). Walker et al. (2014) compared amount of wine poured into wine glasses of different shapes and sizes, for self-defined typical servings, and found 12% more wine was poured into wider glasses than narrower ones of the same capacity, but no difference for wine glasses of different sizes. De Visser and Birch (2012) found that increasing cup size for wine (150 ml *vs* 250 ml) and beer (340 ml *vs* 570 ml) led to increased alcohol units poured for both self-defined usual servings and alcohol units (‘standard drinks’).

Six further studies measure the effect of glassware design on ‘standard drink’ pours. Three studies by Wansink and Van Ittersum (2003, 2005) measured pours of single shots of spirits, finding pours were 3–30% larger in short-wide *vs* tall-narrow glasses (capacity both 355 ml). White et al. (2003) investigated amount of alcohol poured among college students, for ‘standard drinks’ of beer, straight shots – i.e., single serving of spirits – and mixed drinks – i.e., spirit served with a mixer. Participants poured each standard drink into glasses of different sizes. The amount poured was generally higher than a ‘standard drink’, an effect that increased in magnitude with increasing cup size, for all drink and glass types. In a follow up study, White et al. (2005) asked participants to pour standard drinks (beer, straight shots, mixed drinks, and wine) into cups of various sizes (three per drink type). Increasing cup size led to increased volume poured for beer, mixed drinks, and wine. There was an effect of cup size on volume poured for shots in shot glasses, though it was non-linear: there was a U shaped relationship, with less poured into the middle-sized cup. Extending these findings to a Singaporean sample, Zandy et al. (2013) found that increasing cup size led to increased volume pours of ‘standard drinks’ (30 and 220 ml, for shots and beer respectively, based on Singapore Health Promotion Board), for both beer and liquor.

Five studies report the impact of glass design on set volumes (i.e., not standard-drinks or self-defined servings). Chen and Lee (2019) found between 7% and 27% more was poured into larger *vs* smaller glasses of different shapes, and that between 10% and 17% more was poured into tall-slender *vs* short-wide glasses, when participants were asked to pour either 100 ml or 200 ml. Four studies report on glass shape and pouring to drink midpoints. In two studies, when asked to pour to the glass midpoint (165 ml for a 330 ml capacity glass), participants poured ∼14 ml less into outward-sloped tumblers than straight-sided ones (Langfield et al., 2018, 2020), though there was no evidence of a difference between inward-sloped and straight-sided glasses in the former study (Langfield et al., 2018). In a follow up study using stemmed 165 ml glasses, there was no evidence of a difference in estimates of the midpoint (82.5 ml) poured into straight-sided wine flutes and outward-sloped martini coupes, though the direction of the effect was the same (Langfield et al., 2020). Troy and colleagues also found that when estimating midpoints (284 ml) for pint glasses (568 ml), ∼45 ml less was poured into outward-sloped and ∼15 ml less into tulip glasses than straight-sided ones, though – as found by Langfield et al. (2018) – there was no evidence of a difference between inverted and straight-sided glasses.

Three studies highlight that the effects of glassware design (shape and size) on amount poured for set portions may vary with features of the pouring task and nature of the instructions. Caljouw & van Wijck (2014) measured volume of lemonade poured in ‘drink’ and ‘shot’ portions, poured into glasses of different shapes (short-wide *vs* tall-narrow, both 300 ml). There was an interaction between glass shape and drink portion, such that when pouring shots, more was poured in short-wide glasses, but when pouring drinks, more was poured into the tall-narrow glasses. Chen et al. (2017) found that while glass size (large *vs* small) and shape (tall-narrow *vs* short-wide) did influence amount poured for a set portion, the direction of these effects depended on viewing angle, with the direction of the effects reversing when poured at 0 and 30 degrees *vs* 60 and 90 degrees. Chandon and Ordabayeva (2009) measured amount of alcoholic and non-alcoholic drinks poured into various outward-sloped and straight-sided glasses. They found that ‘supersizing’ – pouring three times the volume, *vs* ‘downsizing’ – pouring a third of the volume, reversed the effect of glass shape on amount poured. In particular, more was poured into outward-sloped glasses when supersizing, but the opposite was true when downsizing.

## II. Impacts of glassware design on micro-drinking behaviours

Searches were conducted for experimental studies manipulating glassware (size, shape, and fullness) and measuring human drinking behaviour (total drinking time, sip size, number of sips, sip and interval durations, and drinking trajectory), for alcoholic and non-alcoholic drinks. As for macro-drinking behaviours, observational studies and literature reviews were excluded, and studies were only included if they measured drinking behaviours (not online or via self-report).

### i. Total drinking time

One factor related to the amount consumed, for food at least, is speed of consumption (for a review see Robinson et al., 2014). Quicker eating rates may increase *ad libitum* consumption through one or more of several processes, including lower levels of satiation (e.g., Andrade et al., 2008) and decreased orosensory exposure to the food – the time the food spends in the mouth (de Graaf, 2011). It is plausible that the speed at which drinks are consumed may also influence, or be a proxy for, the total amount consumed. Thus, exploring the conditions under which people consume drinks more quickly – for example, depending on the glass used – may inform why people consume more or less overall. Five studies have investigated the effect of glass shape and size on time taken to consume alcoholic and non-alcoholic drinks, described below.

The effect of glass shape (outward-sloped *vs* straight-sided) on drinking speed has been investigated in three studies. Attwood et al. (2012) found that individuals consumed 340 ml of beer 60% more slowly from straight 340 ml, compared with outward-sloped 340 ml, beer glasses, although no differences were found for a soft drink, or for smaller (170 ml) portions. Glass fullness predicted total drinking time, with full glasses (larger portions) consumed more slowly than half-full glasses (smaller portions). These authors attributed their key finding – that glass shape influenced total drinking time for alcohol – to titration of drinking rate based on biased perception of volumes, with greater bias for outward-sloped glasses due to the nonlinear relationship between height and volume. (See later section on perception for more discussion of this perceptual mechanism). A second study investigated the effect of glass shape – outward-sloped, straight-sided, and inward-sloped tumblers – on total drinking time, using a soft drink (Langfield et al., 2018). In contrast to Attwood and colleagues’ findings (2012), drinking was about 20% slower from the straight-sided glass than the outward-sloped glass for a soft drink. Although drinking from the inward-sloped glass was also faster than from the straight-sided glass, wide confidence intervals suggested no meaningful difference. A third study compared drinking speed for a soft drink (the same as Langfield et al., 2018) from outward-sloped and straight-sided tumblers. There was no evidence or trend to suggest a difference in overall drinking time (Langfield et al., 2020).

A further study investigated the impact of glass shape on total drinking time of an alcoholic cocktail, using straight-sided glasses of different shapes (narrow/tall *vs* short/wide), and measuring drinking in a semi-naturalistic bar-laboratory setting (Cliceri et al., 2018). Participants consumed the 150 ml cocktail about 7% slower from the tall/narrow glass than the short/wide one, although there was no statistical evidence that this difference was meaningful. It is worth noting that straws were used in both conditions, which may have masked differences in drinking afforded by sipping from glasses directly (see section on Affordance).

Glass size has also been investigated in the context of drinking speed. Zupan, Pechey et al. (2017) explored the effect of wine glass capacity on total drinking time in a laboratory setting. Based on previous evidence that larger wine glasses elicited higher sales of wine (Pechey et al., 2016), Zupan and colleagues predicted that wine is consumed more quickly from larger glasses, keeping serving size constant. Contrary to predictions, consumption was 18% slower from larger than smaller wine glasses (370, 250 ml respectively).

### ii. Sip size

There is some evidence from studies on eating behaviour that show that larger portion sizes lead to larger bite sizes (Almiron-Roig et al., 2015) and that eating with large bite sizes increases how much is consumed, alongside an underestimation of the amount consumed – a possible mechanism underlying increased consumption from larger portion sizes (Bolhuis et al., 2013; Hollands et al., 2015). One study has directly manipulated sip size to examine the effect on the amount of a drink that is drunk. Weijzen et al. (2009) investigated the impact of manipulating sip size on the volume of orangeade consumed by giving participants small (5 g) and large (20 g) sips, delivered via a tube in their mouths. Participants self-administered the drink using a pump to initiate each sip, and decided when to terminate drinking. Although the drinking behaviour was highly artificial in nature, the study showed an increase in volume consumed of 20% and 40% when the drink was delivered in larger sip sizes, for sugar-free and sugar-sweetened beverages respectively. Taken together, this evidence suggests that understanding the conditions under which people consume with smaller sips may be important in understanding why people may consume less overall. Three studies have measured sip sizes for non-alcoholic drinks taken from glasses of different sizes and shapes, described below.

Two studies report effects of glass size on sip size, albeit with some caveats. Lawless et al. (2003) found individuals took sips that were about 15% larger from cups with 600 ml *vs* 150 ml capacity, although cup size was confounded with portion size to keep fullness constant. This means it is not clear which variable(s) – portion size, cup size, or both – drove increased sip size. A second study manipulated the nature of drinking – i.e., whether drinking was ‘instructed’ (participants were given a series of cups and instructed to sip from each) or ‘natural’ (participants were given a glass of water without explicit instructions while completing a screening interview) – to determine the impact on sip size (Bennett et al., 2009). The aim of this study was to inform swallowing assessment procedures in clinical settings – which often require patients to take sips – for example, in patients with dysphasia – disordered swallowing. A large effect was found: sip sizes were four times larger in the natural phase compared with the instructed phase (24 ml *vs* 6 ml). However, portion size, as well as cup size, varied between these conditions (from 20 to 50 ml in the instructed tasks to 200 ml in the natural task), meaning larger sips may have been driven by any of these factors – portion size, cup size, instructions – alone or in combination.

A third study reports the effect of glass shape on sip size. Langfield et al. (2020) recorded sip sizes taken from straight-sided wine flutes and outward-sloped martini coupes, with the primary aim being to measure lip muscle activity (see later section on Affordance). Participants placed their drink on concealed weighing scales in between sips, allowing for covert measurement of sip size. Sips were 17% smaller when taken from straight-sided glasses *vs* outward-sloped ones.

### iii. Number of sips

Number of sips may be a proxy for sip size, especially when a set portion is consumed. That is, a drink drunk in fewer sips can be said to have been consumed with larger gulps – on average – than an identical drink drunk in more sips. Six studies have counted number of sips taken to consume alcoholic and non-alcoholic drinks.

In five studies where participants consumed a set portion of drink at their own pace, numbers of sips were explored. Cliceri et al. (2018) compared number of sips taken from a 150 ml portion of cocktail served straight-sided glasses of different shapes (narrow/tall *vs* short/wide). While slightly more sips were taken from the tall-narrow glass, there was no statistical evidence to support that the difference was meaningful. Attwood et al. (2012) compared numbers of sips taken from full (340 ml) and half-full (170 ml) portions of beer and lemonade, served in 340 ml outward-sloped and straight-sided glasses. Incorporating all the data, there were main effects of glass shape and fullness, such that more sips were taken from straight-sided glasses than outward-sloped ones, and more sips taken from full portions than half-full portions. In two studies, there was no evidence that mean sip size – calculated by dividing total amount consumed (330 ml) by number of sips – differed between straight-sided glasses and outward-sloped glasses (Langfield et al., 2018; Langfield et al., 2020) and between straight-sided and inward-sloped glasses (Langfield et al., 2018). Zupan, Pechey et al. (2017) found no evidence that consuming wine in a larger or smaller glass led to differences in number of sips taken to consume a 175 ml portion.

In another study, participants tasted and rated four drinks served in identical glasses during a bogus taste test, and sips were subsequently coded from video recordings (Langfield et al., 2020). While total number of sips did not differ, when expressed as a proportion of total amount consumed (the primary outcome measure, which varied between participants), mean sip size was smaller from straight-sided glasses than outward-sloped ones.

### iv. Sip and interval durations

Four studies have examined sip and interval durations from glasses of different sizes, shapes, and fullness. Zupan, Pechey et al. (2017) found shorter average sip durations for wine consumed in larger *vs* smaller capacity wine glasses. Attwood et al. (2012) found that individuals tended to have longer intervals between sips from the straight *vs* outward-sloped glasses – when sipping full (340 ml) portions – for beer but not lemonade. These authors also found that glass fullness predicted total sip and interval duration, with longer total sipping and inter-sip time from full (340 ml) glasses than half-full (170 ml) ones. In two studies, there was no evidence that glass shape predicted sip or interval duration, for 330 ml soft drink served in straight-sided *vs* outward-sloped glasses (Langfield et al., 2018; Langfield et al., 2020) or straight-sided *vs* inward-sloped glasses (Langfield et al., 2018).

### v. Drinking trajectory

One further micro-drinking behaviour that may differ by glassware design is drinking trajectory within a standardised period – i.e., the dynamic pattern of drinking over time. Here, instead of comparing summaries of micro-drinking behaviours – for example, mean sip size or total number of sips – these micro-drinking behaviours are considered over time within one drinking episode. Studies on eating behaviour have identified ways to monitor dynamic changes in consumption over time, using covert weighing scales which record weights at regular intervals during eating episodes (e.g., ‘Universal Eating Monitor’, Kissileff et al., 1980; ‘Mandometer ®’, Zandian et al., 2009). This continuous measurement allows researchers to plot participants’ cumulative food intake curves, which can be characterised as ‘decelerated’ or ‘linear’ (e.g., Kissileff et al., 1982; Pudel, 1971; Westerterp-Plantenga et al., 1991; Zandian et al., 2009; Zandian et al., 2012). Decelerated eating would be characterised by more rapid consumption at the beginning, such that more is consumed in the first half of the eating episode, while a linear trajectory would be characterised by a more constant pace. It is possible that studies on drinking may also distinguish different drinking trajectories, and determining the conditions under which more ‘decelerated’ or ‘linear’ patterns are present may be informative. Two studies report on the impact of glassware design on drinking trajectory (cumulative intake over time).

Cliceri et al. (2018) plotted consumption over time, and found that drinking from a short, wide glass was more decelerated than drinking from a tall, narrow glass. This decelerated pattern was characterised by a larger volume consumed in the first half of the drinking period. Although a decelerated pattern of consumption was common in this study – only 30% had an accelerated pattern – a greater proportion of individuals drinking from the short, wide glass (81%) showed this pattern, as compared to those drinking from the tall, narrow glass (60.4%).

In exploratory analyses, Langfield et al. (2018) found longer initial, and shorter final, sip durations from the outward-sloped glass, which contrasted with the straight-sided glass, for which the opposite pattern was true. These long initial sip durations may have been proxies for large initial gulps due to the relatively full, outward-sloped glass, though it is not possible to determine trajectory (consumption over time) from sip and interval durations alone. In a follow up study, Langfield et al. (2020) extended these findings by measuring cumulative intake over time (with measures of intake obtained from images of the drinks, as in Cliceri et al., 2018). In this study, there was a difference in drinking trajectory between glass shapes: a more decelerated pattern of consumption was observed from outward-sloped glasses, as compared to straight-sided ones.

## III. Hypothesised mechanisms for impacts of glassware design on drinking behaviours

To optimise and better understand effects of glassware design on consumption (micro- and/or macro- drinking behaviours), it is useful to consider plausible underlying mechanisms as targets for such optimisation. In the following sections, two distinct but not exclusive sets of mechanisms are presented: perception and affordance (see Figure 1 for logic model).

### i. Perception

There is a wealth of evidence concerning the effect of a drink’s container on how the drink is perceived, including ratings of flavour, liking of the drink, and volume perception (Spence & Van Doorn, 2017; Spence & Wan, 2015). Drinks can taste different depending on the shape of a glass. For example, beer may taste fruitier and more intense when served in curved compared with straight-sided glasses (Mirabito et al., 2017). Identical wines have been perceived to be different wines, depending on the shape of the glass in which they were served (Spence, 2011). Satisfaction with the amount of a drink consumed has been shown to be higher when it was served in a tall-narrow glass than when served in a short-wide one (Cliceri et al., 2018). Perceived appropriateness of a drink’s container may also influence liking for the drink (Raudenbush et al., 2002), as well as how much people are willing to pay for alcoholic drinks (Wan et al., 2015). Container design may also influence volume consumed via perceived unit costs, such that drinks in larger containers might be perceived to be less expensive, per unit of volume, as compared to drinks in smaller containers (Wansink, 1996).

For perception of volume, the ability to judge liquid volumes may vary with glass shape and size for wine glasses (Pechey et al., 2015; Walker et al., 2014), glass shape for tumblers and hi-ball glasses (e.g., Wansink & Van Ittersum, 2003, 2005), as well as glass shape (outward-sloped *vs* straight-sided) for both beer glasses (Attwood et al., 2012; Troy et al., 2018) and tumblers (Langfield et al., 2018; Langfield et al., 2020).

**Figure 2.**
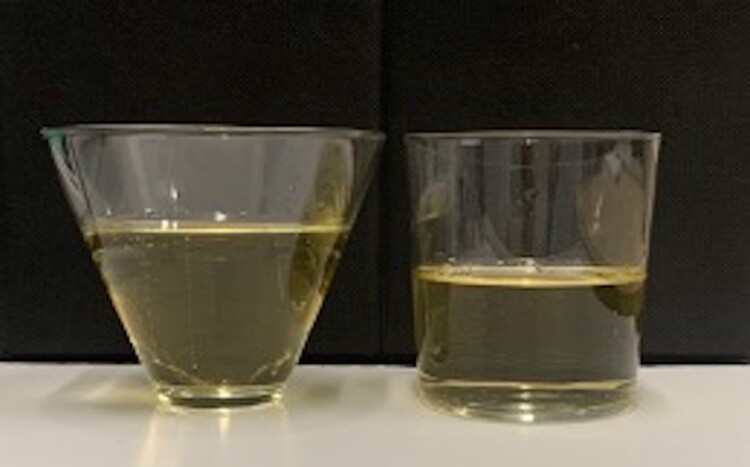
Image to depict the midpoints of 330 ml portions in outward-sloped and straight-sided glasses, as in Langfield et al. (2018) and Langfield et al. (2020).

Specifically, several studies have explored the effect of glass shape on ability to estimate drink midpoints. When comparing straight-sided *vs* outward-sloped glasses, research shows that individuals underestimate the midpoint for outward-sloped glasses to a greater degree than for straight-sided ones, with midpoints underestimated by between 7% and 30% for outward-sloped glasses and 2% and 6% for straight-sided glasses (Attwood et al., 2012; Troy et al., 2018; Langfield et al., 2018, 2020; see Figure 2 for example glasses filled half-way, as in Langfield et al., 2018, 2020).

Bias in midpoint estimation has been examined using both virtual (e.g., Attwood et al., 2012; Troy et al., 2018) and real-life (e.g., Langfield et al., 2018, 2020; Troy et al., 2018) drink pouring tasks. These biases are consistent with conflating height with volume, or an ‘elongation effect’, such that volumes that are taller are perceived as greater (e.g., Raghubir & Krishna, 1999). The elongation effect, and differences in perception of volume found for outward-sloped vs straight-sided glassware, may be driven by a failure to assess the multiplicative impact of changing more than one dimension simultaneously (e.g., object height and width). Individuals may focus on one dimension – such as height – and thus fail to adjust for width (e.g., see Chandon & Ordabayeva, 2009; Krider et al., 2001). The elongation effect has also been found to vary with portion size, or relative fullness of the glass, and may be reversed when pouring large drinks, as opposed to shots (Caljouw & van Wijck, 2014). Use of height as a cue to volume begins at a young age. Seminal experiments by Piaget showed that children aged 2–7 were generally unable to ‘conserve’ the liquid poured from one short-wide container to a tall-narrow one, perceiving the identical volumes differently, depending on the glass shape (e.g., Piaget, 1967).

How a drink is *perceived* – including preferences for drinks, subjective ratings of flavour, and ability to estimate volume – may be one mechanism through which the design of a glass impacts drinking. However, relatively few studies have directly examined whether these *subjective* perceptions of drinks and glassware translate into tangible differences in *objectively* measured drinking behaviours. Indeed, there may be a disconnect between subjective perceptions and objective drinking behaviours. For example, Chandon and Ordabayeva (2017) found participants to be more accurate when estimating decreasing – as opposed to increasing – quantities, although this asymmetry was reduced when pouring quantities, as opposed to estimating numerically. A further study on eating behaviour found self-reported preference for one food item over another predicted selection of that food item, but did not predict the amount consumed – measured using covert video recordings (Iborra-Bernad et al., 2012). Taken together, these studies suggest perceptions, subjective ratings, and even selections, may not always be accurate predictors of behaviour.

As previously discussed, four studies report drinkers underestimate the mid-point of a glass to a greater extent for outward-sloped compared with straight-sided glasses (Attwood et al., 2012; Langfield et al., 2018, 2020; Troy et al., 2018). Midpoint bias might, in turn, impact drinking behaviour, via titration of consumption based on false information about amount consumed. That is, if midpoints are underestimated, drinkers will have consumed more than half of their drink when they reach their perceived midpoint. This might speed up consumption, if drinking is titrated based on biased midpoints. The relationship between midpoint bias and drinking behaviour has been explored in four studies.

Attwood et al. (2012) found a trend towards a positive association between the degree of perceptual midpoint bias and rate of consumption (*r* = 0.15). This might reflect an underpowered analysis or other mechanisms contributing to the differences in drinking speed. In three subsequent studies, no association was found between midpoint bias and drinking time (*r* = 0.01, −0.09), or midpoint bias and amount consumed (*r *= −0.03), for consumption of soft drinks (Langfield et al., 2018, 2020). If midpoint bias is an important determinant of drinking behaviour, clear midpoint labels on outward-sloped glasses may slow consumption relative to unmarked outward-sloped glasses. Troy et al. (2017) found a trend suggesting that labelling the half-way point slowed drinking speed relative to unmarked glasses, but the confidence intervals were wide and also consistent with faster drinking. Taken together, these findings suggest that factors other than perception – and in this case volume perception – may be driving effects of glass shape on drinking speed more strongly, at least for outward-sloped and straight-sided glasses.

Thus, although there are many studies on the impact of the drink container on how the drink is perceived, further studies are warranted to determine the extent to which perceptual effects, including bias in volume perception, as well as subjective ratings such as for liking and flavour can explain variation in drinking behaviours.

### ii. Affordance

An alternative or additional mechanism that may underlie the effects of glassware design on drinking behaviours is *affordance*, described by Gibson (1979) as ‘what it (*an object or the environment*) offers to the animal, what it provides or furnishes, either for good or ill’ (p.127). These ideas were later popularised by Norman, a student of Gibson’s, in ‘The Psychology of Everyday Things’ (later ‘The Design of Everyday Things’), and were applied to objects in our environment that were seemingly poorly designed, failing to *afford* the appropriate behaviour (Norman, 1988, 2013). The primary difference between the two conceptualisations is that, for Norman, the key insight is in how actors can *design* environments that afford behaviours more easily, while Gibson was more interested in how actors perceive existing environments (McGrenere & Ho, 2000). Further, for Norman, affordances can make actions easier or more difficult (rather than simply exist or not exist, as implied by Gibson; McGrenere & Ho, 2000).

In the context of drinking behaviours, there are a number of ways affordance might be a useful concept. Two studies have characterised the ecological affordances of *alcogenic* environments such as pubs, through observation and interviews (Hill, Foxcroft, et al., 2018; Hill, Pilling, et al., 2018). One example of an affordance identified by these researchers was faster drinking rates when individuals could not place their drinks on tables. That is, a pattern of drinking – in this case, increased drinking rate – was apparently afforded by the wider drinking environment, and in particular by a lack of a ‘put-on-able’ surface (Hill, Foxcroft, et al., 2018, p. 459).

#### Glassware design and affordance

**Figure 3.**
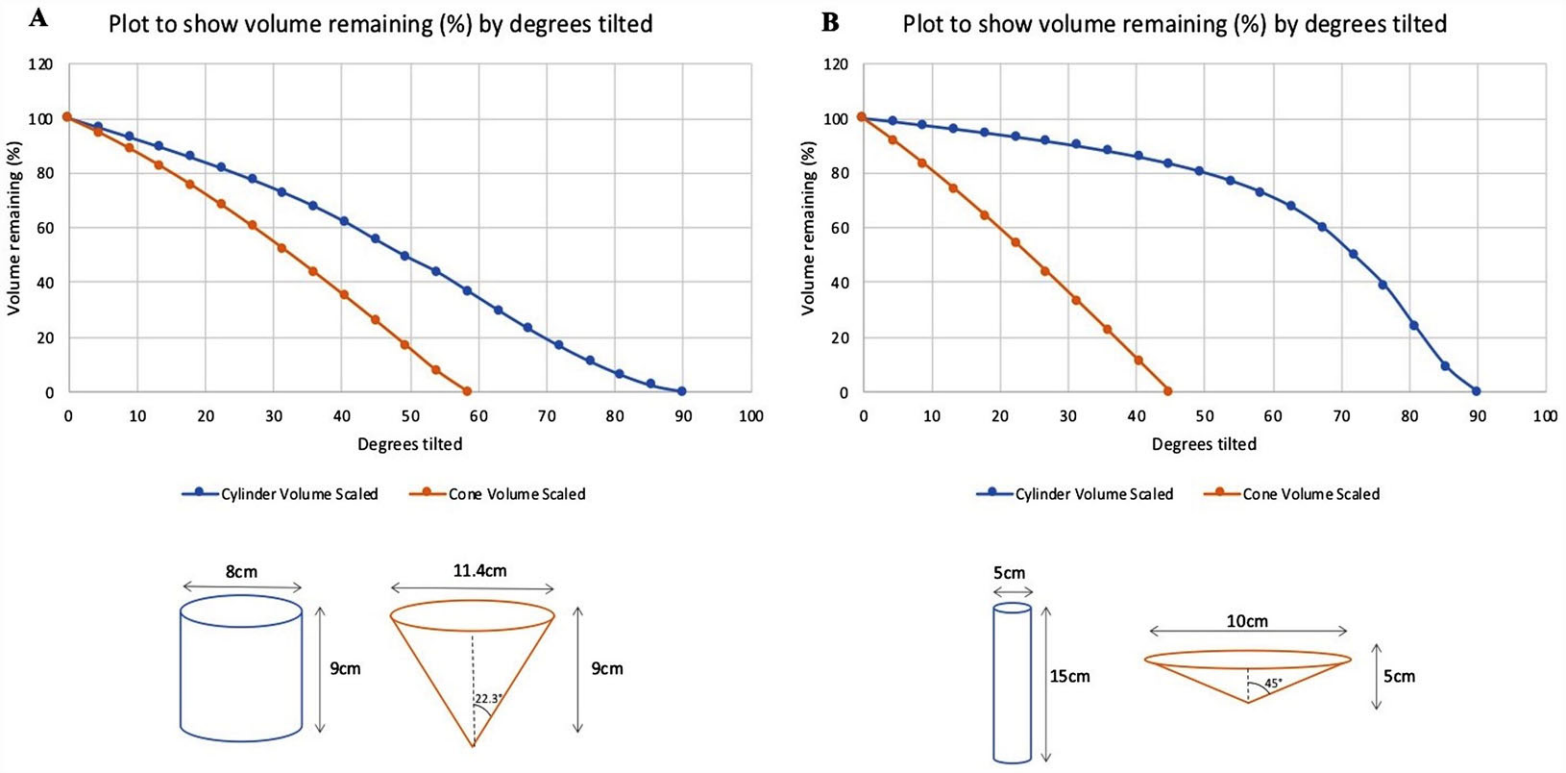
Plots to show affordance by glass shape of volume remaining (%) by angle of tilt. ‘A’ illustrates the relationship with glass dimensions as used by Langfield et al., 2018; Study 1 (Langfield et al., 2020). ‘B’ illustrates the relationship with more extreme dimensions.

Broadly, then, characteristics of a drinking environment might be said to *afford* an increase or a decrease in drinking, for example, by the nature of the room layout. The glass from which a drink is consumed may also afford more or less of this drink being drunk, depending on its design. Indeed, some of the basic properties of the design of a glass, such as its size, shape, and fullness, might afford specific patterns of drinking behaviours. For example, the flow of liquid when a glass is tilted may differ depending on the shape of the glass. This can be observed when comparing the flow of liquid from an outward-sloped compared with a straight-sided glass. When full, outward-sloped glasses – which resemble truncated cones – appear to spill easily. They require relatively less tilt than full straight-sided glasses – which resemble cylinders – to pour out the same volume. Figure 3 plots volume poured by pouring angle, for cones and cylinders, for (a) tumblers with the same dimensions as those used by Langfield and colleagues (Langfield et al., 2018; Study 1; Langfield et al., 2020), and (b) more extreme versions (Study 2 & 3; Langfield et al., 2020). For more information on how these plots were obtained, see Supplementary Information.

When drinking, volume tipped into the mouth can thus be influenced by the simple affordance of different glass shapes. Less tilt – and potentially less effort – is required to tip the same amount of liquid into the mouth from an outward-sloped glass than a straight-sided one (see Figure 3(A,B)). This affordance of liquid pouring by pouring angle from different glass shapes can shed light on some of the findings on glass shape and drinking behaviours. For example, tilting a full outward-sloped (conical) glass to the lips to extract a sip may afford a larger initial sip, when compared to tilting a full straight-sided (cylindrical) glass. This might contribute to a more decelerated pattern of consumption – characterised by a larger amount consumed in the first half of consumption from outward-sloped glasses – as found in a recent study (Langfield et al., 2020).

An additional affordance by glassware design might involve embouchure – the extent of lip pursing – when sipping. Glasses of different designs may afford greater (or less) pursed embouchures, leading to smaller (or greater) sized sips, resulting in less (or more) being consumed. Using facial electromyography, one study found greater muscle activity used in the lips when participants sipped through a straw, as compared to sipping from a spoon or a cup (Murray et al., 1998). Glasses of different shapes and sizes may also cue differences in embouchures. This was explored in a recent study (Langfield et al., 2020). Using facial electrodes attached to the upper and lower lips to measure embouchure, this study found increased lip muscle activity, indicative of more pursed embouchures, when participants sipped from straight-sided wine flutes than from outward-sloped martini coupes. Sips were also smaller from wine flutes, but there was limited evidence from this preliminary study that embouchures mediated this effect. Thus there is some evidence to support the hypothesis that different glass shapes afford different embouchures, but further studies are required to validate these preliminary findings in a study with greater power to detect smaller effects than was possible in this preliminary study.

A final affordance of glassware design on consumption is the affordance of volume poured from larger *vs* smaller glasses. Larger glasses afford larger pours, by nature of the maximum capacity of the container, and this may contribute to increased consumption from larger glasses. Indeed, in their analysis of the effects of wine glass size on wine sales from bars and restaurants, Pilling et al. (2020) found that larger wine glasses led to increased purchasing of wine in the restaurants settings but not in bars. One explanation that is offered by the authors is that wine is more commonly served by the bottle in restaurants, which allows consumers to free-pour their wine, so larger wine glasses may have afforded larger pours, and thus, increased consumption.

## IV. Typology of drinking behaviours

## Discussion

### Summary of review findings

The first aim of this paper was to review evidence on the impact of glassware design on consumption of alcoholic and non-alcoholic drinks (Aim I). The review reveals a paucity of evidence on the effects of glassware design on drinking behaviour and in particular on volume consumed. Together, the evidence indicates potential effects of glassware design – including size and shape (and relatively less evidence on fullness) – on drinking behaviours using outcome measures that may be correlates or proxies of consumption, such as amount poured and amount purchased. For example, there are some consistent effects of glassware design (size and shape) on amount poured, across a range of drink types, sizes, portions, and shapes. Taken together, the research suggests that more is poured into larger than smaller glasses, short-wide than tall-narrow glasses, and straight-sided than outward-sloped glasses, though these effects may vary depending on how much is being poured (e.g., Caljouw & van Wijck, 2014), whether pouring is ‘supersizing’ or ‘downsizing’ (Chandon & Ordabayeva, 2009), viewing angle (e.g., Chen et al., 2017), and whether the pour is self-defined (e.g., Knibb et al., 2018) or a specific volume (e.g., Wansink & Van Ittersum, 2003).

One particular area for future research concerns the extent to which macro-drinking behaviours, such as amount poured, may act as proxies for consumption. Studies might involve measurements of amounts poured, as well as number of drinks, to explore possible compensatory effects. For example, if less is poured into and consumed from smaller glasses, at what size might the use of smaller glasses increase consumption through compensatory behaviour? This is also important as it will aid the design of glassware which strikes the right balance, addressing the issue of when a glass becomes ‘too small’, such that compensatory behaviours are elicited.

The review also highlights the growing evidence on the impacts of glassware design on micro-drinking behaviours (Aim II). This includes research on sip size, with some preliminary evidence suggesting larger sips taken from larger (and wider-rimmed) cups. Studying the micro-structure of drinking – using the typology presented here as a starting point – has the potential to develop understanding of these effects and in particular whether and how much they link to volume consumed.

On the basis of the evidence reported in this review, the methods used to measure different micro-drinking behaviours may merit refinement. For example, studies involving sipping behaviours often use crude measures, including *mean* sip size (e.g., Langfield et al., 2018), or *total* number of sips (e.g., Zupan, Attwood et al., 2012; Pechey et al., 2017). A more promising method may be using a more dynamic approach to studying drinking behaviour – including measuring ‘drinking trajectories’ or sip sizes over time. These approaches provide a more precise estimate of the dynamics of consumption (i.e., the drinking trajectory), illustrating how drinking behaviour might change over the course of a drinking episode. While eating behaviour has been characterised by a quadratic curve (e.g., Kissileff et al., 1982), the two studies reported here use quadratic (Cliceri et al., 2018) and cubic (Langfield et al., 2020) curves to characterise drinking over time. The shape of these curves may also differ depending on conditions (such as glass shape; see Langfield et al., 2020). Thus, measuring both micro- and macro- drinking behaviours using both static and dynamic measures will provide a more complete picture of drinking which – in turn – may achieve a greater understanding of the effects of glassware design on consumption, though the significance of these drinking trajectories for overall consumption remains unstudied.

### Elucidating mechanism: affordance and perceptual effects

In addition to increasing the quality and quantity of evidence on how glassware design affects drinking behaviours, studies are needed to advance understanding of the mechanisms by which glassware design might affect consumption. The third aim of this paper was to highlight two potential mechanisms: perceptions (including hedonic ratings such as subjective ratings of liking and flavour, as well as volume judgments) and affordance (Aim III). The logic model we presented in Figure 1 summarises these proposed mechanisms of action. When evaluating these mechanisms, it is important to identify *i.* how glassware design influences perceptions of a drink and *ii.* how these perceptions influence drinking behaviour, as well as *iii.* how glassware design affords certain behaviours such as liquid flow and embouchures and *iv.* how these in turn influence drinking behaviour. The evidence presented in this review begins to address these questions. There is, for example, much evidence for *i.* but less for *ii.* There is little evidence for *iii.* and *iv*. but what evidence there is appears promising.

Importantly, these are neither the only mechanisms by which drinks containers affect consumption, nor are any mechanisms likely to operate alone. For example, a glass might influence liquid volume judgments, which in turn influences volume poured. This, at one level, could influence how much is consumed. Additionally, the same glass might cue large initial sips due to the physical affordances of the glass and its rim diameter and slope when tipped, as well as the embouchure it elicits. These large initial gulps might then speed up drinking and lead to an increase in amount consumed. Future studies could attempt to isolate each mechanism, to determine whether the effect on drinking behaviour remains. For example, opaque glasses with different shapes, sizes, and fullness could be used, to limit visual perception of drink volumes and possibly midpoint bias, which has been found to vary by glass shape (e.g., Attwood et al., 2012; Langfield et al., 2018). To ‘limit’ the role of affordance via lip embouchures, which may vary depending on glass shape (e.g., Langfield et al., 2020), future studies might provide straws (which likely elicit the same lip embouchure regardless of the glass being sipped from). Should the effect of glassware design on measures of consumption remain, this might cast some doubt as to the importance of embouchure as a potential mechanism.

Continuing to situate these mechanisms of affordance and perception within studies on glassware design and drinking behaviours is helpful, to advance our understanding of the effects. However, as suggested by Hollands et al. (2016), in the context of behaviour change research, exploring mechanism may only be ‘fundamentally a means to an end’ (p. 390). Ultimately, elucidating the underlying mechanisms driving the effects of glassware design on consumption is helpful primarily to inform the design of better interventions, which in this case, may aim to reduce consumption of health-harming drinks.

### Typology of drinking behaviours

The final aim of this paper was to develop a preliminary typology of drinking behaviours. It is clear when reviewing the existing evidence, that there has been a lack of consistency and clarity in reporting on drinking behaviours. For example, small-scale drinking behaviours – which reflect the micro-structure of a drinking episode – have been variously described as ‘micro-drinking behaviours’ (e.g., Langfield et al., 2018; Zupan, Pechey, et al., 2017), ‘drinking topography’ (Attwood et al., 2012; Foy & Simon, 1978; Troy et al., 2017), ‘kinetics of consumption’ (e.g., Giboreau, 2018) or, borrowing from the eating behaviour literature, ‘oral processing behaviours’ (e.g., Ferriday et al., 2016; Krop et al., 2018), ‘intrameal eating and drinking patterns’ (Bellisle & Le Magnen, 1981; Warner & Balagura, 1975), and meal ‘micro-structure’ (e.g., Almiron-Roig et al., 2015; Doulah et al., 2017). The typology presented here contrasts ‘micro-drinking behaviours’ with ‘larger-scale’ drinking behaviours (consumption and proxies for it), which we term ‘macro-drinking behaviours’. See Table 1 for typology.

**Table 1.** Typology of macro- and micro- drinking behaviours.

	Behaviour	Definition	Measurement	Example references
**Macro**	**Amount consumed**	Amount that is consumed (e.g., ml). Also referred to as *ad libitum consumption, total intake, volume ingested, volume consumed etc.*	Measure the volume consumed (ml), for example by weighing the drink(s) before and after consumption.	Kersbergen et al. (2018); Langfield et al. (2020)
	**Amount purchased**	Amount that is purchased. This can be used as a proxy for amount consumed (particularly in field studies with no direct measurement of behaviour).	Calculate the amount spent (e.g., £), and transform into volume (ml) purchased.	Pechey et al. (2016); Clarke et al. (2019); Troy et al. (2015)
	**Amount poured**	Amount that is poured (e.g., ml). This can be a self-defined serving, a specific volume (e.g., ‘standard drink’). Can be used as a proxy for consumption (or, combined with number of drinks to calculate b amount consumed)	Measure the volume poured (ml), for example by weighing the drink(s) before and after the pour, or by using measuring cylinders.	Wansink and Van Ittersum (2003); Knibb et al. (2018); Langfield et al. (2018)
	**Number of drinks**	Number of drinks consumed. This can be calculated for a given consumption occasion (e.g., how many times people pour themselves another glass) or across consumption occasions (e.g., number of drinks per week). Can be used as a proxy for consumption (or, combined with amount poured or served to calculate amount consumed).	Count the number of drinks served, poured, purchased, or consumed. For example, observe and count the total number of beverages (e.g., pints of beer) sold over an evening.	***No studies identified***
**Micro**	**Total drinking time**	Time taken to consume a drink (e.g., min). Also referred to as *speed of consumption, drinking speed, drinking rate, total time drinking etc.*	Measure the time it takes to consume a given drink (e.g., with a stopwatch, or from coding video recordings).	Attwood et al. (2012); Zupan, Pechey, et al. (2017); Troy et al. (2017); Brunstrom et al. (2000); Langfield et al. (2018)
	**Sip size**	Size of sip (ml). Also known as sip volume, bolus volume.	To measure exact sip sizes, hidden weighing scales can be used, or participants can be asked to spit into a cup. To determine average sip size, divide total volume consumed by number of sips, which can be counted from video recordings of drinking sessions.	Langfield et al. (2020); Lawless et al. (2003); Bennett et al. (2009); Langfield et al. (2018)
	**No. of sips**	Number of sips taken to consume a drink. Also known as *sip frequency*.	Can count number of sips from video recordings of drinking sessions.	Attwood et al. (2012); Zupan, Pechey, et al. (2017); Troy et al. (2017)
	**Sip rate**	Rate of sipping (e.g., ml/s).	Mean sip size is divided by total time spent drinking, to give sip rate.	Tomaszewski et al. (1980)
	**Sip duration**	Time taken to drink a sip. Related concepts are *orosensory exposure time* and *total bout duration* (although these are often operationalised as a *total* – i.e., across all sips – while sip durations often refer to an average based on individual sips).	Can measure sip durations using video recordings of drinking sessions, and coding when each sip is initiated, and when it ends.	Attwood et al. (2012); Zupan, Pechey, et al. (2017); Troy et al. (2017); Brunstrom et al. (2000); Langfield et al. (2018)
	**Interval duration**	Length of time between sips. Also known as *inter-sip interval / idle time / inter-bout interval*	Can measure interval durations using video recordings of drinking sessions, and coding when each sip ends, and when the next is initiated.	Attwood et al. (2012); Troy et al. (2017); Brunstrom et al. (2000); Langfield et al. (2018)
	**Drinking trajectory**	Dynamic pattern of drinking rate across the drinking period. Also known as *dynamic drinking rate, drinking rate across the drinking period.*	Extract height information from video recordings and map height of liquid:glass to volume, based on a model of volume by height ratios. Alternatively use a hidden weighing scale (for example, in a drinks coaster), to plot the weight of the glass periodically on a graph. Helpful to plot drinking trajectories within a standardised period, if comparing between individuals. Some example drinking trajectories include: ‘*S’ shaped (cubic); accelerated (exponential); decelerated (logarithmic); linear.*	Cliceri et al. (2018); Langfield et al. (2020)
	**No. of swallows**	Number of swallows taken during the consumption of a drink. Note – may differ from number of sips – e.g., a large sip may be swallowed in two gulps.	Microphone attached to throat can be used, to identify timing of swallow (and thus the number of swallows in a given time period).	Bennett et al. (2009)

Note: Macro-drinking behaviour: measures of drinking outcomes involving consumption, or proxies for consumption. Micro-drinking behaviour: a form of short-term influence on drinking. Also known as: drinking topography, oral processing behaviours, microstructure of drinking behaviour.

Using this typology as a framework and starting point for understanding the micro-structure of a drinking episode may harness important insights for developing interventions aimed at reducing consumption. Indeed, as mentioned previously, this level of detail might illuminate *how* an intervention works to reduce intake. For example, certain glass designs may cue less consumption *via* smaller sips, or *via* slower-paced consumption characterised by long intervals in between sips. This level of detail in describing a drinking episode may also give clues to important effects on drinking behaviours that may not be captured by a ‘macro’ measure of drinking in a given study.

### Limitations

When manipulating glassware design (shape, size or fullness), it is rare that the manipulation isolates a particular design feature, without other features confounding with these features. For example, when varying glass shape, in attempting to keep glass capacity constant, glass height (Attwood et al., 2012), and rim diameter (Langfield et al., 2018, 2020), can vary. Similarly, when attempting to keep glass height constant, capacity may vary (which can lead to differences in fullness, given the same portion served; as in Langfield et al., 2018). It is similarly difficult to determine the causes of some of the effects, where both portion size and glass size are varied (e.g., Kersbergen et al., 2018; Lawless et al., 2003). Thus, a limitation of this body of research is that it can be difficult to determine the exact feature of a drinks container that influences consumption.

Further, many of the studies reported in this review were conducted in laboratory settings which, though advantageous for elucidating mechanism, may be limited in reflecting intervention effects in real world settings. Relatively few studies took place in real-life settings such as pubs and restaurants (but see Clarke et al., 2019; Pechey et al., 2016; 2017; Troy et al., 2015). It should be noted that measuring food or drink consumption directly is difficult in field studies, with selection and purchasing data used as a proxy for the amount consumed. Nonetheless, these field studies are crucial to estimate effect sizes – at a population level – of any intervention involving drinks containers such as glassware. Such settings include many contextual effects that may influence behaviour which cannot be reproduced in laboratory settings (Giboreau, 2018). Laboratory studies of drinking behaviour often involve solitary drinking (e.g., Attwood et al., 2012; Langfield et al., 2018, 2020; Troy et al., 2017), potentially failing to reflect social nature of much drinking, especially common for consumption of alcohol. Semi-naturalistic laboratories set up to appear like restaurants and bars provide greater ecological validity than traditional laboratory settings (e.g., Cliceri et al., 2018; Kersbergen et al., 2018) although still less than that of a field setting. Future studies should also examine consumption of multiple drinks, to investigate how drinking behaviours change over longer periods, which may, again, be more reflective of real-life drinking (especially for alcohol). As discussed previously, smaller glasses might lead to less drink poured for a single glass. However, it is possible that compensatory strategies lead people to consume more overall – for example, by consuming a higher number of drinks over a longer drinking period. Here, measuring number of drinks consumed, as well as amount poured, could be informative.

This review summarises evidence on studies using alcoholic and non-alcoholic drinks. One limitation is that in most cases it isn’t possible to compare the effects of glassware design on consumption of different drink types due to the lack of evidence. However, there are potential differences between these drink types, including the effects of alcohol on decision making, and motivations behind consumption, with quantity of alcohol consumed likely more salient than quantity of soft drinks consumed, in certain contexts. Given these possible differences, further research here would be particularly beneficial.

### Implementation of interventions involving glassware design

There are several routes to implementing an intervention involving glassware design to reduce consumption of health-harming drinks. These include voluntary action, regulation, and legislation. Given possible barriers to change, including public acceptability of interventions and potential cost, researchers should continue to strive for evidence of the effectiveness and likely parameters for any given intervention involving glassware design. Such parameters include drink-type (e.g., alcoholic *vs* non-alcoholic), drinking context (e.g., bar, restaurant, or home, with drinks pre-served or self-served), and drinking pattern when multiple drinks are consumed. Effect size estimates, generated from multiple studies, are also required to make predictions about the possible impact of an intervention at a population level. Together, these factors would help to form a robust evidence base which is required for any regulation-based policies, especially given that these policies are likely to be resisted by producers and retailers of the drinks targeted by the policy (Freudenberg, 2014; Pomeranz & Brownell, 2014). Perhaps one of the key challenges to implementation is to ‘change minds’ about changing behaviour, with a focus on changing *environments* (in this case, glassware), not *individuals* (Marteau, 2018).

### Conclusion

There is a paucity of evidence on the impact of the design of glassware on drinking behaviours, although several studies suggest it might affect how much is consumed, with some evidence for several candidate mechanisms. The provisional typology presented here and analysis of the limited existing evidence provides a starting point for subsequent research in order to generate a coherent body of evidence that can advance understanding of the impact of glassware design on macro-drinking behaviours – consumption and its proxies – as well as micro-drinking behaviours that contribute to this including sip size. To identify glassware design features worth targeting for intervention, research needs to continue a focus on the effects of glassware design on amount consumed and on micro-drinking behaviours, which may be important in understanding the mechanisms driving any overall consumption effects. The robustness of this research will be enhanced by more valid and granular measures of macro- and micro- drinking behaviours, in both laboratory and field settings. In addition, to optimise these effects, the underlying mechanisms warrant further exploration. This review highlighted perceptions and affordances as two possibilities, though neither exclusive nor exhaustive. The evidence summary presented here – including the logic model and typology – provides an initial basis for building an evidence base on a promising set of interventions to reduce consumption of alcoholic and non-alcoholic drinks that harm health.

## Supplementary Material

Supplemental Material
