# Supplementary material for: Glassware design and drinking behaviours: a review of impact and mechanisms using a new typology of drinking behaviours
Source: Health Psychol Rev. 2020 Nov 18;16(1):81–103. doi: 10.1080/17437199.2020.1842230 (PMC8884253; doi:10.1080/17437199.2020.1842230)
Supplement: Supplemental Material [file RHPR_A_1842230_SM4547.pdf]

Supplementary Information for:

Glassware design and drinking behaviours: a review of impact and mechanisms using a new typology of drinking behaviours

Tess Langfield, Rachel Pechey, Mark A. Pilling, Theresa M. Marteau

University of Cambridge

Supplementary Information:

Search strategy and terms

Tables S1 & S2: Details of all studies included in review

Determining volume poured when glasses are tilted

Search strategy

An electronic literature search was completed on 2 Sep 2020, to source relevant papers on the impact of glassware design (size, shape, fullness) on macro- and micro- drinking behaviours. Eligibility criteria included: experimental design (i.e. non-observational or literature reviews), measuring human drinking behaviour (i.e. not measured virtually, online, or using self-reported drinking), with researcher assignment to condition (i.e. glassware design features manipulated, not participant self-selected, in between-subjects designs) or presentation order (within-subject designs).

Databases searched: **MEDLINE** and **PsycInfo**.
All searches combined: 671
Removing duplicates: 607
Papers meeting eligibility criteria: 23
Snowball searching and personal communication: 4
Total papers included in review: 27

MEDLINE Database (including In-Process & Other Non-Indexed Citations 1946 to September 2, 2020)
Date searched = 2nd September 2020
Papers returned = 311

((glass* or drinkware or cup or container) adj9 (size* or capacit* or portion* or volume* or shape* or fullness))
AND
(drink* or drunk* or consum* or sale* or sold or purchas* or sip* or taste* or pour* or drink frequency or drink number or number of drinks)

PsycInfo Database (from 1835-2020)
Date searched = 2nd September 2020
Papers returned = 360

(glass* OR drinkware OR cup OR container)
AND
(size* OR capacit* OR portion* OR volume* OR shape* OR fullness)
AND
(drink* OR drunk* OR consum* OR sale* OR sold OR purchas* OR sip* OR taste* OR pour* OR drink frequency OR drink number OR number of drinks)

Additional papers

Snowball searching revealed an additional three papers (de Visser & Birch, 2012; Bennett et al., 2009; Kersbergen et al., 2018). Personal communication with authors revealed one trial pre-registration (Brocklebank, 2019). All included papers are listed in Table S1 and S2, including study details and findings.

Table S1. Experimental studies investigating the impact of glassware design (size, shape, fullness) on macro-drinking behaviours

**Author (date)**	**Study setting**	**Glassware manipulation** (size, shape, fullness)	**Drinks served** (including portion size)	**Outcome measure** (drinking behaviour)	**Study finding**
**Amount consumed**					
Kersbergen et al. (2018)	Study 1 Semi-naturalistic laboratory study; N = 114; between-subjects design<br><br>Study 2 Field study – bar; N = 166*; between-subjects design<br><br>Both UK<br><br>*N.B. after excluding participants aware of alcohol intake measure - n = 164	Glass size*<br><br>Study 1: ‘standard’ wine 310ml vs ‘smaller’ wine 250ml; ‘standard’ beer 530ml vs ‘smaller’ beer 370ml<br><br>Study 2: ‘standard’ wine 245ml vs ‘smaller’ wine 195ml; ‘standard’ beer 568ml vs ‘smaller’ beer 379ml<br><br>*N.B. actual manipulation – portion size –confounded with glass size	Alcohol (wine, beer, cider)<br><br>Study 1: wine served by the glass in ‘standard’ 165ml or ‘reduced’ 125ml portions; beer and cider served by the glass in ‘standard’ 460ml or ‘reduced’ 345ml portions.<br><br>Study 2: wine served by the glass in ‘standard’ 175ml or ‘reduced’ 125ml portions; beer and cider served by the glass in ‘standard’ 568ml or ‘reduced’ 379ml portions.	Amount consumed (in UK alcohol units)*<br><br>*N.B. though consumption was measured directly in the laboratory study (through weighing the glasses before and after consumption), to measure intake in the bar, drinking was measured through observation by researchers	Study 1 - reducing the serving size of alcohol led to reduction in alcoholic units consumed (*p*=.02)<br><br>Study 2 - reducing the serving size of alcohol led to reduction in alcoholic units consumed (*p*=.001)<br><br>N.B. Study 1 analysis adjusted for covariates; Study 2 analysis unadjusted
Langfield et al. (2020)	Study 2 Laboratory study; N = 72; between-subjects design; UK	Glass shape: outward-sloped martini coupes (260ml) vs straight-sided wine flutes (210ml)	Non-alcoholic (passionfruit drink), 660ml served, divided into four 165ml portions	Amount consumed (ml) in bogus taste test	72ml less was consumed from straight-sided glasses than outward-sloped glasses (*p*=.022)
Raghubir & Krishna (1999)	Study 4 Field study – classroom; N = 16; within-subjects design; USA	Glass shape (taller vs shorter; both 296ml)	Non-alcoholic (cola), portion size NR	Amount consumed (ml) when asked to taste the two drinks	11% more was consumed from the taller glass (*p*<.0001).
**Amount purchased**					
Pechey et al. (2016)	Field study – 1 establishment with bar and restaurant; multiple-treatment reversal design; UK	Wine glass size<br><br>‘standard’ 300ml vs ‘larger’ 370ml and ‘smaller’ 250ml	Alcohol (wine), served by the glass (125ml, 175ml), bottle (750ml) or carafe (500ml, 1000ml)	Daily volume of wine purchased (ml)	Daily volume of wine purchased was 9% higher from larger wine glasses than standard glasses (*p*=.015)<br><br>No difference between smaller and standard glasses (*p*=.63)
Pechey et al. (2017)	Field study – 2 bars; multiple-treatment reversal design; UK	Wine glass size<br><br>Bar 1: ‘standard’ 370ml vs ‘smaller’	Alcohol (wine), served by the glass (125ml, 175ml,	Daily volume of wine purchased (ml)	Bar 1: Daily volume of wine purchased was 11% higher from

		300ml and ‘larger’ 510ml<br><br>Bar 2: ‘smaller’ 300ml vs ‘larger’ 510ml	250ml), bottle (750ml)		larger wine glasses than standard glasses (95%CI 1%, 21%)<br><br>No difference between smaller and standard glasses (smaller, 95% CI -5%,20%)<br><br>Bar 2: No difference between larger and smaller glasses (larger 95%CI 12.6%,11.9%)
Clarke et al. (2019)	Study 1 & 2<br>Field study – restaurant<br><br>Study 3<br>Field study – bar<br><br>Study 4<br>Field study – bar<br><br>All multiple-treatment reversal design; UK	Wine glass size<br><br>Study 1: ‘smaller’ 290ml vs ‘standard’ 350ml and ‘larger’ 450ml<br><br>Study 2: ‘smaller’ 290ml vs ‘standard’ 350ml and ‘larger’ 450ml<br><br>Study 3: ‘standard’ 350ml vs ‘smaller’ 290ml and ‘larger’ 450ml<br><br>Study 3: ‘standard’ 350ml vs ‘smaller’ 290ml and ‘larger’ 450ml	Alcohol (wine), served by the glass (125ml, 175ml, 250ml – the latter, Study 3 and 4 only), bottle (750ml), and carafe (500ml, 1000ml – carafe, Study 1 & 2 only)	Daily volume of wine purchased (ml)	Study 1: Daily volume of wine purchased was 13% higher from standard wine glasses than smaller glasses (95% CI 2%, 24%)<br><br>Study 2: Daily volume of wine purchased was 6% higher from standard wine glasses than smaller glasses (95% CI − 1%, 15%)<br><br>Study 3: Daily volume of wine purchased was 21% higher from larger wine glasses than standard wine glasses (95% CI 9%, 35%)<br><br>Study 4: No evidence of replication of Study 3 found (− 7%, 95% CI − 16%, 3%)<br><br>No meaningful differences with other glass comparisons
Troy et al. (2015)	Field study – 3 public houses, UK	Beer glass shape<br><br>Outward-sloped (284ml, 568ml) vs straight-sided (290ml, 585ml)	Alcohol (beer, cider), served by the glass (568ml, 284ml)	Monetary takings (not including food), over each weekend period*<br><br>*N.B. primary outcome was feasibility; overall monetary takings included other purchases	Monetary takings were 24% lower on weekends when straight-sided glasses were used, though confidence intervals crossed zero (95% CI: -77%, 29%)

Brocklebank (2019)	Field study pre-registration – 24 public houses; multi-period crossover trial with randomisation; UK	Beer glass shape<br><br>Straight-sided (568ml, 284ml) vs usual curved* glassware (568ml, 284ml)<br><br>*width of the curved glassware not consistent from bottom to top	Alcohol (beer, ale, cider), served by the glass (568ml, 284ml)	Volume of lager, ale and cider purchased (ml)	N/A – study underway
**Amount poured**					
Wansink & van Ittersum (2003)*<br><br>*N.B. Several papers from Brian Wansink have been retracted or are under suspicion.	Study 1<br>Field study – fitness camp cafeteria; N = 97 children at a fitness camp; between-subjects design<br><br>Study 2<br>Field study – cafeteria; N = 89 adults; between-subjects design<br><br>Both USA	Study 1 & 2<br>Glass shape: short-wide tumbler (659ml) vs tall-narrow highball (659ml)	Study 1 & 2<br>Non-alcoholic (juice)	Study 1 & 2 - Volume poured (ml), in a freely poured self-serving	Study 1 – 74% more juice was poured into short-wide glasses than tall-narrow ones (*p*<.05)<br><br>Study 2 – 19% more juice was poured into short-wide glasses than tall-narrow ones (*p*<.05)
Knibb et al. (2018)	Study 1<br>Laboratory study; N = 126; between-subjects; UK	Glass shape: short-wide vs tall-narrow (capacity both 300ml)	Alcohol* in a vodka bottle<br><br>*N.B. all drinks poured were non-alcoholic	Volume of alcohol poured (ml), when asked to pour their usual serving of spirit	No evidence of difference in poured servings between short-wide and tall-narrow (*p*=.748)
Walker et al. (2014)	Laboratory study; N = 73; mixed design; USA	Glass shape: Wider red wine glass vs narrower white wine glass<br><br>Glass size: Smaller vs larger wine glass<br><br>Capacities NR	Alcohol (red and white wine)	Volume of wine poured (ml), when pouring a typical serving	More poured into wider glass than narrower one (12%, p<.05)<br><br>No effect of glass size on amount poured (p>.05)
De Visser & Birch (2012)	Laboratory study; N = 125; within-subjects design; UK	Cup size<br><br>Wine (250ml, 150ml);<br><br>Beer (570ml, 340ml)	Alcohol* in 750ml red wine bottle, 660ml beer bottle and 375ml vodka bottle<br><br>*N.B. all drinks poured were non-alcoholic, dyed to appear the correct colour	Amount poured (in UK alcohol units), when pouring “usual self-serving” and a “standard drink” for wine, beer, and vodka	Units poured increased with cup size, for wine and beer, for both “unit” and “usual serving” pours (*p* NR)
Wansink & van Ittersum (2003)*<br><br>*N.B. Several papers from Brian Wansink have been retracted or are under suspicion.	Study 3<br>Field study – bars; N = 45 bartenders; between-subjects design, USA	Glass shape: short-wide tumbler (355ml) vs tall-narrow highball (355ml)	Alcohol (gin, vodka, rum and whiskey; from 1500ml bottles)	Volume of alcohol poured (ml), estimating a standard 1.5oz (44.4ml) serving	Study 3 – 27% more poured into short-wide glasses than tall-narrow ones (*p*<.01)
Wansink & van Ittersum (2005)	Study 1<br>Laboratory study; N = 198; between-subjects design	Glass shape: short-wide tumbler (355ml) vs tall-narrow highball (355ml)	Alcohol* (gin, vodka, rum and whiskey; from 1500ml bottles)	Volume of alcohol poured (ml), estimating a standard 1.5oz (44.4ml) serving	Study 1 – 30% more poured into short-wide glasses than tall-narrow ones (*p*<.001)

*N.B. Several papers from Brian Wansink have been retracted or are under suspicion.	Study 2<br>Field study – bars; N = 86 bartenders; between-subjects design<br><br>Both USA		*N.B. all drinks poured were non-alcoholic		Study 2 –20.5% more poured into short-wide glasses than tall-narrow ones (*p*<.0001)
White et al. (2003)	Laboratory study; N = 106; within-subjects design, USA	Cup size:<br><br>Shot glass (44.4ml) and cups (88.7ml, 177ml)<br><br>Mixed drink cups (177ml, 296ml, 355ml)<br><br>Beer cups (473ml, 710ml, 946ml)	Alcohol* in a clear glass 750ml liquor bottle (shot/mixed drinks) and 9464ml cooler with a spigot (beer)<br><br>*N.B. all drinks poured were non-alcoholic	Volume of alcohol poured (ml), estimating a standard 1.25oz (37.0ml) serving of liquor, and 12oz (355ml) serving of beer	Increasing cup size led to increased volume pours of water for 'shots' in shot glasses (*p*<.001), 'shots' in mixed drink glasses (*p*<.001), and 'beer' in beer glasses (*p*<.001)
White et al. (2005)	Laboratory study; N = 133; within-subjects design, USA	Cup size:<br><br>Shots (88.7ml, 266.ml, 355ml)<br><br>Mixed drink (266ml, 355ml, 473ml)<br><br>Beer (473ml, 591ml, 946ml)<br><br>Wine (355ml, 473ml, 591ml)	Alcohol* in a clear glass 750ml liquor bottle (shot/mixed drinks) and 9464ml cooler with a spigot (beer), and a clear glass 750ml wine bottle (wine)<br><br>*N.B. all drinks poured were non-alcoholic, dyed to appear the correct colour	Volume of alcohol poured (ml), estimating a standard 1.25oz (37.0ml) / 1.5oz (44.4ml) serving of liquor, 12oz (355ml) serving of beer, 4oz (118ml) / 5oz (148ml) serving of wine	Increasing cup size led to increased volume pours for 'beer' in beer cups, 'wine' in wine cups, and 'shots' in mixed drink glasses (linear trends, *p*s<.05).<br><br>There was a non-linear trend for shots poured in shot cups (*p*<.001).
Zandy et al. (2013)	Laboratory study; N = 105; within-subjects design, Singapore	Cup size<br><br>Shots (44.4ml, 88.7ml, 207ml)<br><br>Beer (473ml, 651ml, 946ml)	Alcohol* in 1000ml pitchers (beer) and a glass 1000ml liquor bottle (shot)<br><br>*N.B. all drinks poured were non-alcoholic, dyed to appear the correct colour	Volume of alcohol poured (ml), estimating a standard 30ml serving of liquor, and 220ml serving of beer	Increasing cup size led to increased volume pours for 'beer' *p*<.0001, and 'shots' *p*<.0001.
Chen & Lee (2019)	Laboratory study; N = 60; mixed design; Taiwan	Glass elongation (short-wide vs tall-narrow wine glasses and tumblers)<br><br>Glass size (large vs small wine glasses and tumblers)<br><br>NB this study also manipulated lighting level and liquid colour	Alcohol (red wine) and non-alcohol (water)	Volume poured (ml) when estimating set portions (100ml or 200ml, depending on glass)	Pouring into tumblers, 27% more was poured into large vs small ones (*p*<.001).<br><br>Pouring into wine glasses, 7% more was poured into large vs small glasses (*p*<.05).<br><br>Pouring into tumblers, 17% more was poured into tall-slender vs short-wide glasses (*p*<.001).

					Pouring into wine glasses, 10% more was poured into the tall-slender vs short-wide glasses (*p*<.001).
Langfield et al. (2018)	Laboratory study; N = 162; between-subjects design; UK	Glass shape: outward-sloped (400ml) vs straight-sided (400ml) vs inward-sloped (440ml) tumblers	Non-alcoholic (carbonated apple drink), 330ml serving, and 660ml in jug	Volume poured (ml) when estimating 165ml portions (i.e. midpoint of full 330ml portion)	Less poured into outward-sloped glass than straight-sided ones (*p*<.001). No evidence of difference between inward-sloped and straight-sided (*p*=.82)
Langfield et al. (2020)	Study 1 Laboratory study; N = 200*; between-subjects design; UK<br><br>*N.B. final sample 198 participants	Glass shape: outward-sloped vs straight-sided tumblers (capacity both 400ml)	Non-alcoholic (carbonated apple drink), 330ml serving, and 660ml in jug	Study 1 Volume poured (ml) when estimating 165ml portions (i.e. midpoint of full 330ml portion)	Less poured into outward-sloped glass than straight-sided ones (*p*<.001)
" "	Study 2 Laboratory study; N = 72; between-subjects design; UK	Glass shape: outward-sloped martini coupes (260ml) vs straight-sided wine flutes (210ml)	Non-alcoholic (passionfruit drink), 165ml serving, and 500ml in jug	Study 2 Volume poured (ml) when estimating 82.5ml portions (i.e. midpoint of full 165ml portion)	No evidence of difference in poured estimates between outward-sloped and straight-sided (*p*=.531)
Troy et al. (2018)	Study 2 Laboratory study; N = 96; within-subjects design; UK	Glass shape: straight-sided vs outward-sloped vs tulip vs inverted (capacity all 568ml)	Non-alcoholic (water in a jug)	Volume poured (ml) when estimating 284ml portions (i.e. midpoint of the full 568ml portion)	Less poured into outward-sloped and tulip glasses than straight-sided ones (*ps*<.001). No evidence of a difference between inverted and straight-sided (*p*=.95).
Caljouw & van Wijck (2014)	Study 1 Laboratory study; N = 42; within-subjects; The Netherlands	Glass shape: short-wide vs tall-narrow (capacity both 300ml)<br><br>NB this study also manipulated pouring instructions	Non-alcoholic (lemonade), 1.5 litre jug	Volume poured (ml), when pouring a "drink" and a "shot"	Interaction between glass shape and instruction (*p*<.001): when pouring shots, volume poured is greater in short-wide than tall-narrow glass, but when pouring drinks, the opposite is true
Chen et al. (2017)	Laboratory study; N = 50; mixed design; Taiwan	Glass shape (square vs round, both 230ml)<br><br>Glass elongation (short-wide tumbler vs tall-narrow highball, both 230ml)<br><br>Glass size (large vs small tumbler	Non-alcoholic (water coloured to appear as juice, 500ml in jug)	Volume poured (ml) when estimating 150ml (glass elongation; shape) and 200ml (glass size)	No differences found for square vs round, at any viewing angle.<br><br>Glass elongation When pouring at 0° & 30°, less was poured into tall-slender glasses than short-wide ones (*ps*<.01), but when pouring at

		of same shape, 250ml vs 370ml)<br><br>NB this study also manipulated viewing angle (0°, 30°, 60°, 90°)			60° & 90°, the opposite was true (*ps*<.001).<br><br>Glass size<br>When pouring at 0° & 30°, less was poured into large glasses than small ones (*ps*<.006), but when pouring at 60° & 90°, the opposite was true (*ps*<.007).
Chandon & Ordabayeva (2009)	Study 3<br>Laboratory study; N = 47; between-subjects design*; France<br><br>*NB Randomisation to condition NR	Glass shape (outward-sloped ("3D") vs straight-sided ("1D"); capacities identical – vodka glasses both 100ml, cocktail glasses both 250ml, and infant drinks 20ml*).<br><br>* Data was pooled and rescaled across product variations<br><br>NB This study also manipulated pouring instructions (i.e. downsize vs supersize)	Alcoholic (vodka, cocktail) and non-alcoholic (infant medicine), in opaque jugs	Volume poured (expressed as a multiple of initial dose), when asked to pour 'three times' the served dose (supersizing), or 'a third' of the served dose (downsizing)	When asked to pour three times the initial serving, more was poured into outward-sloped glasses than straight-sided ones (*p*<.005).<br><br>When asked to pour a third of the initial serving, less was left in outward-sloped glasses than straight-sided ones (*p*<.003).

Note. Exact p values given unless where not reported.

Table S2. Experimental studies investigating the impact of glassware design (size, shape, fullness) on micro-drinking behaviours

**Author (date)**	**Study setting**	**Glassware manipulation** (size, shape, fullness)	**Drinks served** (including portion size)	**Outcome measure** (drinking behaviour)	**Study finding**
**Total drinking time**					
Attwood et al. (2012)	Laboratory study; N = 160*; between-subjects design; UK<br><br>*N.B. useable data available for 159 participants	Glass shape (beer glasses: 340ml outward-sloped vs 340ml straight-sided)<br><br>Glass fullness (half - 170ml vs full - 340ml portions)	Alcohol (beer) and non-alcoholic (lemonade), 340ml and 170ml servings	Total drinking time (min), measured from coded video recordings of participants drinking at their own pace	Stratified analyses on full (340ml) portions – slower drinking from straight-sided glasses for beer (*p*= .007) but not lemonade (*p*=.78)<br><br>Incorporating all data, main effect of portion: slower drinking of full portions than half-full portions (*p*<.001)
Langfield et al. (2018)	Laboratory study; N = 162; between-subjects design; UK	Glass shape: outward-sloped (400ml) vs straight-sided (400ml) vs inward-sloped (440ml) tumblers	Non-alcoholic (carbonated apple drink), 330ml serving	Total drinking time (min), measured from coded video recordings of participants drinking at their own pace	Slower drinking from straight-sided glasses than outward-sloped glasses (percentage difference = 21.4%), *p*=.048
Langfield et al. (2020)	Study 1 Laboratory study; N = 200*; between-subjects design; UK<br><br>*N.B. useable drinking time data available for 198 participants	Glass shape: outward-sloped vs straight-sided tumblers (capacity both 400ml)	Non-alcoholic (carbonated apple drink), 330ml serving	Total drinking time (min), measured from coded video recordings of participants drinking at their own pace	No evidence of a difference in total drinking time (*p*=.979)
Cliceri et al. (2018)	Field study – "Living Lab" restaurant; N = 123*; between-subjects design; France<br><br>*N.B. useable data available for 90 participants	Glass shape: short-wide tumbler (200ml) vs tall-narrow highball (220ml)	Alcohol (orange based cocktail), 150ml serving	Total drinking time (sec), measured from coded video recordings of participants drinking at their own pace	Slower drinking from tall-narrow glasses than short-wide glasses, (mean difference = 48 sec), though no statistical evidence that this difference was meaningful (*p*=.168).
Zupan, Pechey, et al. (2017)	Laboratory study; N = 166; between-subjects design; UK	Wine glass size<br><br>'smaller' 250ml vs 'larger' 370ml	Alcohol (red wine), 175ml serving	Total drinking time (sec), measured from coded video recordings of participants drinking at their own pace	Larger wine glasses led to slower drinking times than smaller wine glasses (*p*=.024).
**Sip size**					
Lawless et al. (2003)	Study 1 Laboratory study; N = 100*; within-	Cup size* (150ml vs 300ml vs 600ml capacity)	Non-alcoholic (water, filled to 0.6cm from the	Sip size (ml), measured by asking participants	Larger cups led to increased sip sizes in Group 1 (*p*<.05)

	subjects design; USA<br><br>*N.B. split into two groups – Group 2 (n = 50) contained taller women and shorter men to minimize body size variable predicting sip size	*N.B. to keep fullness constant, cup size was confounded with portion size	top of each of the three cups)	to take a sip and spit into a cup	and Group 2 (*p*<.01)
Bennett et al. (2009)	Laboratory study; N = 32; within-subjects design; Canada	Cup size (exact capacities NR, though largest was 227ml)<br><br>*N.B. actual manipulation – presence/absence of explicit instructions to sip, i.e. natural vs instructed sipping – confounded with cup size and portion size	Non-alcoholic (water, served in portions of 200ml (“uninstructed”), 50ml and 20ml (“instructed”))	Sip sizes (ml) measured by experimenter when drinks placed on hidden weighing scales between sips	Sips were smaller during “instructed” task, i.e. when sipping from smaller cups, as compared to the “uninstructed” task, i.e. when sipping from larger cups *p*<.0001
Langfield et al. (2020)	Study 3 Laboratory study; N = 40; within-subjects design; Australia	Glass shape: outward-sloped martini coupes (260ml) vs straight-sided wine flutes (210ml)	Non-alcoholic (passionfruit drink), 660ml served, divided into four 165ml portions	Sip sizes (ml) measured by experimenter when drinks placed on hidden weighing scales between sips, during adapted bogus taste test	Sip sizes were smaller from straight-sided glasses than outward-sloped ones, *p*<.0001
**Number of sips**					
Cliceri et al. (2018)	Semi-naturalistic laboratory study – “Living Lab” restaurant; N = 123*; between-subjects design; France<br><br>*N.B. useable data available for 90 participants	Glass shape: short-wide tumbler (200ml) vs tall-narrow highball (220ml)	Alcohol (orange based cocktail), 150ml serving	Number of sips, measured from coded video recordings of participants drinking at their own pace	Slightly higher number of sips from tall-slender glass than short-wide glass, though no statistical evidence (*p*=.259)
Attwood et al. (2012)	Laboratory study; N = 160*; between-subjects design; UK<br><br>*N.B. useable data available for 159 participants	Glass shape (beer glasses: 340ml outward-sloped vs 340ml straight-sided)<br><br>Glass fullness (half - 170ml vs full - 340ml portions)	Alcohol (beer) and non-alcoholic (lemonade)	Number of sips, measured from coded video recordings of participants drinking at their own pace	Incorporating all data, main effect of glass shape, with more sips taken from straight-sided glasses (*p*=.016), and a main effect of glass fullness, with more sips taken from full portions (*p*<.001)
Langfield et al. (2018)	Laboratory study; N = 162; between-subjects design; UK	Glass shape: outward-sloped (400ml) vs straight-sided (400ml) vs inward-sloped (440ml) tumblers	Non-alcoholic (carbonated apple drink), 330ml serving	Number of sips (transformed into mean sip size, by expressing as a proportion of total amount consumed – 330ml), measured from coded video recordings of participants	Mean sip sizes were larger from inward and outward-sloped glasses than straight-sided glasses, though no statistically meaningful difference observed (*p*=.057,

				drinking at their own pace	*p*=.13, respectively)
Langfield et al. (2020)	Study 1<br>Laboratory study; N = 200*; between-subjects design; UK<br><br>*N.B. useable number of sips data available for 198 participants	Glass shape: outward-sloped vs straight-sided tumblers (capacity both 400ml)	Non-alcoholic (carbonated apple drink), 330ml serving	Study 1<br>Number of sips (transformed into mean sip size, by expressing as a proportion of total amount consumed – 330ml), measured from coded video recordings of participants drinking at their own pace	Mean sip sizes were larger from outward-sloped glasses than straight-sided glasses, though no statistically meaningful difference observed (*p*=.123)
" "	Study 2<br>Laboratory study; N = 72*; between-subjects design; UK<br><br>**N.B. useable sip data available for 71 participants	Glass shape: outward-sloped martini coupes (260ml) vs straight-sided wine flutes (210ml)	Non-alcoholic (passionfruit drink), 660ml served, divided into four 165ml portions	Study 2<br>Number of sips, measured from coded video recordings of participants tasting and rating four drinks in a bogus taste test<br><br>Also transformed to mean sip size, as a proportion of total amount consumed – the primary outcome measure)	Number of sips did not differ (*p*=.58), though when expressed as a proportion of total amount consumed, mean sip size did differ, with smaller sips from straight-sided glasses (*p*=.017)
Zupan, Pechey, et al. (2017)	Laboratory study; N = 166; between-subjects design; UK	Wine glass size<br><br>'smaller' 250ml vs 'larger' 370ml	Alcohol (red wine), 175ml serving	Number of sips, measured from coded video recordings of participants drinking at their own pace	Number of sips did not differ between smaller vs larger glasses (*p*=.26)
**Sip duration**					
Attwood et al. (2012)	Laboratory study; N = 160*; between-subjects design; UK<br><br>*N.B. useable data available for 159 participants	Glass shape (beer glasses: 340ml outward-sloped vs 340ml straight-sided)<br><br>Glass fullness (half - 170ml vs full - 340ml portions)	Alcohol (beer) and non-alcoholic (lemonade)	Total sip duration (sec), measured from coded video recordings of participants drinking at their own pace	Incorporating all data, main effect of glass fullness, with longer total sip duration from full portions (*p*<.001)
Zupan, Pechey, et al. (2017)	Laboratory study; N = 166; between-subjects design; UK	Wine glass size<br><br>'smaller' 250ml vs 'larger' 370ml	Alcohol (red wine), 175ml serving	Mean sip duration (sec), measured from coded video recordings of participants drinking at their own pace	Mean sip durations were shorter from the larger glass (*p*=.045)
Langfield et al. (2018)	Laboratory study; N = 162; between-subjects design; UK	Glass shape: outward-sloped (400ml) vs straight-sided (400ml) vs inward-sloped (440ml) tumblers	Non-alcoholic (carbonated apple drink), 330ml serving	Mean sip duration (sec), measured from coded video recordings of participants drinking at their own pace	Mean sip durations did not differ between straight-sided vs outward-sloped glasses (*p*=.40)

Langfield et al. (2020)	Study 1<br>Laboratory study; N = 200*; between-subjects design; UK<br><br>*N.B. useable data available for 198 participants	Glass shape: outward-sloped vs straight-sided tumblers (capacity both 400ml)	Non-alcoholic (carbonated apple drink), 330ml serving	Mean sip duration (sec), measured from coded video recordings of participants drinking at their own pace	Mean sip durations did not differ between straight-sided vs outward-sloped glasses (*p*=.19)
**Interval duration**					
Attwood et al. (2012)	Laboratory study; N = 160*; between-subjects design; UK<br><br>*N.B. useable data available for 159 participants	Glass shape (beer glasses: 340ml outward-sloped vs 340ml straight-sided)<br>Glass fullness (half - 170ml vs full - 340ml portions)	Alcohol (beer) and non-alcoholic (lemonade)	Total interval duration (sec), measured from coded video recordings of participants drinking at their own pace	Incorporating all data, main effect of glass shape, with longer total interval duration from straight-sided glasses (*p*=.031), and a main effect of glass fullness, with longer total interval duration from full portions (*p*=.001)<br><br>Stratified analyses on full (340ml) portions – longer total interval duration from straight-sided glasses for beer (*p*=.008), but not for lemonade (*p*=.83).
Langfield et al. (2018)	Laboratory study; N = 162; between-subjects design; UK	Glass shape: outward-sloped (400ml) vs straight-sided (400ml) vs inward-sloped (440ml) tumblers	Non-alcoholic (carbonated apple drink), 330ml serving	Mean interval duration (sec), measured from coded video recordings of participants drinking at their own pace	Mean interval durations did not differ between straight-sided vs outward-sloped glasses (*p*=.68)
Langfield et al. (2020)	Study 1<br>Laboratory study; N = 200*; between-subjects design; UK<br><br>*N.B. useable data available for 198 participants	Glass shape: outward-sloped vs straight-sided tumblers (capacity both 400ml)	Non-alcoholic (carbonated apple drink), 330ml serving	Mean interval duration (sec), measured from coded video recordings of participants drinking at their own pace	Mean interval durations did not differ between straight-sided vs outward-sloped glasses (*p*=.31)
**Drinking trajectory**					
Cliceri et al. (2018)	Semi-naturalistic laboratory study – “Living Lab” restaurant; N = 123*; between-subjects design; France<br><br>*N.B. useable data available for 90 participants	Glass shape: short-wide tumbler (200ml) vs tall-narrow highball (220ml)	Alcohol (orange based cocktail), 150ml serving	Drinking trajectory (i.e. i.“intake pattern” – proportion accelerated or decelerated, and ii. amount consumed at 50% time)	Proportion of decelerated drinkers (relative to accelerated drinkers) larger in short-wide condition (*p*=.035).<br><br>More consumed from short-wide glasses than tall-narrow glasses at

					50% time (*p*=.004).
Langfield et al. (2020)	Study 1<br>Laboratory study; N = 200*; between-subjects design; UK<br><br>*N.B. useable trajectory data available for n = 16 or n = 94 participants, depending on exclusion criteria)	Glass shape: outward-sloped vs straight-sided tumblers (capacity both 400ml)	Non-alcoholic (carbonated apple drink), 330ml serving	Drinking trajectory (i.e. plot cumulative intake (%) over time (%) and determine:<br>i. amount consumed at 50% time (>50% indicative of decelerated pattern)<br>ii. area under individual's drinking curve (higher values indicative of more decelerated pattern)	From cubic models predicting cumulative intake over time, at 50% time, more was consumed from outward-sloped glasses (59-66%) than straight-sided glasses (50-56%), indicative of decelerated pattern<br><br>Areas under drinking curves were larger from outward-sloped glasses, indicative of more decelerated pattern (*ps*<.023)

Note. Exact p values given unless where not reported.

Determining volume poured when glasses are tilted

Outward-sloped glasses – "cone"

We initially consider a simple symmetric verticle cone with the vertex at the bottom and filled with liquid, with height h and radius r. The volume of this cone is given by $V_{cone} = \frac{1}{3}\pi r^2 h$. At the cone is tilted, liquid is lost and the remaining liquid also forms a cone with h given by the perpendicular distance from the vertex to the the remaining level of the fluid. The volume of this oblique cone, no matter the angle of tilt, is given by the same formula. The perpendicular height h can be expressed as a function of the length of the longest side (l) of the cone and the angle (radians) of tilt (δ) and the internal angle of the cone (φ) from verticle as $h = l.\cos\,(\varphi + \delta)$. This formula is used to estimate the volume of liquid remaining as the cone is tilted.

Straight-sided glasses – "cylinder"

We initially consider a simple symmetric verticle cylinder with the vertex at the bottom and filled with liquid, with height h and radius r. The volume of this cone is given by $V_{cylinder} = \pi r^2 h$. As the cone is tilted by an angle ($\theta = \frac{\pi}{2} - \delta$), as seen in Figure S1 below, the remaining liquid initially forms i) a smaller cylinder of height h-x and ii) a half-cylinder of height x. The distance x is given by $\frac{2r}{tan\theta}$. When x=h (second image) then half the volume has been poured, which occurs when $\tan(\theta) = 2r/h$. Let z be height along the bottom after half the liquid has been poured, given by $z = h.tan(\theta)$. The new volume remaining is half the volume of a segment across the cylinder with of height z, and is given by $V_{cylinder} = \frac{1}{2}h(r^2 cos^{-1}\left(\frac{r-z}{r}\right) - (r-z)\sqrt{(2rz - z^2)})$ for the remainder of the pour.

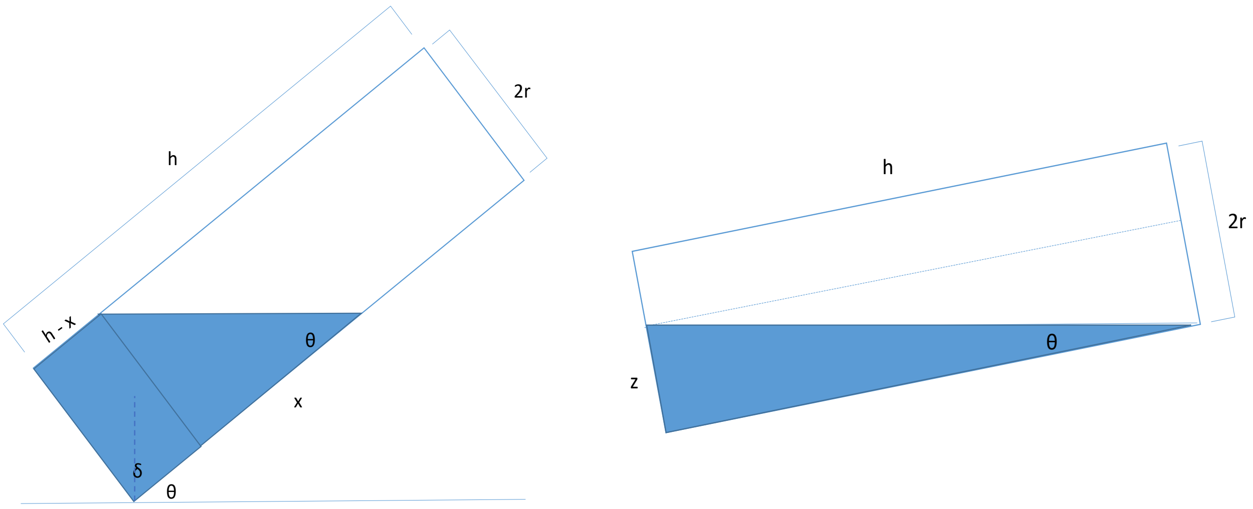

Figure S1. Images to show volumes poured from cylinders, at varying degrees of tilt (δ)
